# ﻿New species of *Mallocybe* (Agaricales, Inocybaceae) from Pakistan, based on morphological and molecular evidence

**DOI:** 10.3897/mycokeys.99.86844

**Published:** 2023-09-07

**Authors:** Malka Saba, Abdul Nasir Khalid, Samina Sarwar

**Affiliations:** 1 Department of Plant Sciences, Quaid-i-Azam University, Islamabad, 45320, Pakistan Quaid-i-Azam University Islamabad Pakistan; 2 Institute of Botany, University of the Punjab, Lahore, Pakistan University of the Punjab Lahore Pakistan; 3 Department of Botany, Lahore College for Women University, Lahore, Pakistan Lahore College for Women University Lahore Pakistan

**Keywords:** Asia, molecular systematics, phylogeny, Pinaceae

## Abstract

Within the family Inocybaceae, many species of *Mallocybe* have been reported, but there are only a few reports of this genus from Pakistan. In this study, six collections of *Mallocybe* were studied by morphological and phylogenetic methods. Phylogenetic analyses, based on sequence data from two different loci (ITS and LSU) using Maximum Likelihood and Maximum Parsimony methods, have been performed to infer species relationships within *Mallocybe*. Results indicated that these six collections encompass two new species of *Mallocybe* i.e. *M.pakistanica* and *M.pinicola*, from Pakistan. Their detailed morphological descriptions and illustrations are also provided. In addition, comparison with morphologically closely-related taxa is also discussed. Previously, only two species of this genus have been recorded from Pakistan and, with this addition, the total number of reported taxa of *Mallocybe* has been raised to four from Pakistan. A key to the described taxa of *Mallocybe* from Pakistan is also provided.

## ﻿Introduction

The Inocybaceae Jülich is a monophyletic family encompassing ectomycorrhizal fungi with worldwide distribution (Matheny et al. 2006) and is estimated to contain 1050 species (Matheny and Kudzma 2019). *Mallocybe* (Kuyper) Matheny, Vizzini & Esteve-Rav was first described as a subgenus of *Inocybe* (Fr.) Fr. (1863:346), but recently, Matheny et al. (2019) elevated *Mallocybe* to one of seven genera in Inocybaceae.

Macroscopically, *Mallocybe* species are recognised by a fibrous or scaly often flattened pileus, a short stipe, ochre, brown or red brown colouration, a cortina, adnate lamellae and absence of a spermatic odour. Microscopically, distinctive characters of this genus include; smooth spores, absence of pleurocystidia, thin-walled without crystals cheilocystidia and necropigment in basidia of fresh and dried specimens (Kuyper 1986; Stangl 1989; Jacobsson 2008; Matheny et al. 2019). This genus is monophyletic and about 58 species of *Mallocybe* have been recorded in Index Fungorum (www.indexfungorum.org) from different regions of Africa, Asia, Australia, Europe, New Zealand and North America (Matheny et al. 2019; Saba and Khalid 2020; Aignon et al. 2021; Mao et al. 2022). However, only two species of this genus are yet known from Pakistan (Ahmad et al. 1997; Saba and Khalid 2020). During the exploration of ectomycorrhizal fungi of Pakistan, we encountered several species of *Mallocybe* which have been described using morphological and molecular analysis (Saba 2016). Amongst them, *Mallocybevelutina* has already been described (Saba and Khalid 2020), while *M.pakistanica* and *M.pinicola* are herein described as new species. Both of these species were found in association with pines in Pakistan.

## ﻿Materials and methods

### ﻿Morphological studies

During an investigation of ectomycorrhizal fungi associated with pine species in Pakistan, basidiomata were collected, described and photographed from the selected sampling sites in the field. Colours were compared to the Munsell Soil Colour Charts (1975) guide. Collections were dried using a food dehydrator (at 39 °C for 7–9 hours). Microscopic characters were observed in the laboratory using hand-cut sections of basidiomata mounted in a 5% aqueous solution of potassium hydroxide (KOH) and of Congo red. Micromorphological analysis and measurements were made, using an Olympus B×40 light microscope with Olympus XC50 digital camera and Microsuite special edition software 3.1 (Soft imaging solutions GmbH). Thirty-five basidiospores were measured from each collection cited. Measurements include the range with extremes provided in parentheses. Q values (length/width ratios) and mean values (average basidiospore length and width) are also provided. Line drawings were made with a Leitz camera Lucida (Wetzlar, Germany). Collections of the newly-described species are deposited at ISL (Quaid-i-Azam University, Islamabad, Pakistan).

### ﻿DNA extraction, PCR amplification and DNA sequencing

Genomic DNA was extracted from a 20 mg piece of dried tissue by a modified CTAB method (Lee et al. 1988). Loci examined during this study included the complete ITS region (ITS1–5.8S–ITS2) of the nuclear ribosomal RNA gene (hereafter ITS) and the first ca. 900 bp of the nuclear 28S rRNA gene (nrLSU).

Primers used for amplification were: ITS1F (Gardes and Bruns 1993) and ITS4 (White et al. 1990) for ITS and LR0R and LR5 for nrLSU (Vilgalys and Hester 1990). The amplification reaction mixture contained 2.5 µl Econo buffer, 0.5 µl dNTPs, 1.25 µl each primer, 0.125 µl Econo Taq, 14.375 µl of deionised water and 5 µl of template DNA. The thermal profile of PCR for ITS was initial denaturation at 94 °C for 1 min.; then 35 cycles of denaturation at 94 °C for 1 min, annealing at 53 °C for 1 min and extension at 72 °C for 1 min; and final extension at 72 °C for 8 min. For nrLSU: 94 °C for 2 min; then 40 cycles of 94 °C for 1 min, 52 °C for 1 min and 72 °C for 1:30 min; and 72 °C for 5 min.

PCR products were run on 1% agarose gel, stained with ethidium bromide and bands were visualised under a UV transilluminator. Amplified PCR products of the ITS region were sent for purification and bidirectional sequencing to Macrogen (Republic of Korea). PCR products of 28S were purified using QIAquick PCR purification kit (Qiagen, Stanford, California) as per manufacturer’s guidelines and sequencing reactions were performed using the Big Dye Terminator v.3.1 Cycle Kit (Life Technologies, Carlsbad, California). Sequencing was carried out using the same primers as those used for PCR.

### ﻿Sequence alignment

Sequences were manually edited and assembled in BioEdit v.7.2.6 (Hall 1999). Generated ITS sequences were trimmed with the conserved motifs 5’–CATTA– and –GACCT–3’ (Dentinger et al. 2011) and the alignment portion between these motifs was included in subsequent analyses. BLASTn searches were performed in NCBI GenBank.

### ﻿BLASTn Results

In the BLASTn search, based on ITS sequences, *Mallocybepakistanica* had the highest sequence identity (94.76%) with *Mallocybemegalospora* (Stangl & Bresinsky) Matheny & Esteve-Rav. HQ604786 (unpublished sequence). *Mallocybepinicola* had the highest sequence identity (93.86%) with type sequence of *Mallocybesiciliana* (Brugaletta, Consiglio & M. Marchetti) Brugaletta, Consiglio & M. Marchetti NR_164583 (Brugaletta et al. 2017). In the BLASTn search, based on LSU sequences, *Mallocybepakistanica* had the highest sequence identity (98.23%) with *Mallocybe* sp. BK 6-June-97-24 (MN178541) which is not yet published. *Mallocybepinicola* had the highest sequence identity (98.90%) with Inocybeaff.malenconii (MN178539).

### ﻿Phylogenetic analysis

Closely-related sequences were retrieved from NCBI GenBank (https://www.ncbi.nlm.nih.gov/genbank/), following Vauras and Larsson (2011) and Saba and Khalid (2020). We also added sequences from GenBank of the close hit after initial BLAST to construct the phylogeny using the combined ITS+nrLSU dataset. Finally, we included *M.arthrocystis* (Kühner) Matheny & Esteve-Rav. as outgroup, following Vauras and Larsson (2011) and Saba and Khalid (2020).

To estimate the placement and phylogenetic relationships of the new species, Maximum Likelihood (ML) and Maximum Parsimony (MP) analyses of the concatenated ITS+nrLSU datasets were conducted. MP analysis was performed in PAUP* version 4.0b10 (Swofford 2002). All characters were equally weighted and gaps were treated as missing data. Trees were inferred using the heuristic search option with TBR branch swapping and 1,000 random sequence additions. Max-trees were set to 5,000, branches of zero length were collapsed and all parsimonious trees were saved. Clade robustness was assessed using a bootstrap (BS) analysis with 1,000 replicates (Felsenstein 1985). Descriptive tree statistics, tree length (TL), consistency index (CI), retention index (RI), rescaled consistency index (RC) and homoplasy index (HI) were calculated for each maximum parsimonious tree generated. Maximum Likelihood (ML) was performed using RAxML 8.0.14 with a general time-reversible (GTR) model of site substitution including estimation of Gamma-distributed rate heterogeneity (+G) and a proportion of invariant sites (+I) on Abe through the CIPRES Science Gateway (www.phylo.org; Miller et al. 2010). Branch support for ML analysis was determined by 1,000 bootstrap replicates (Hillis and Bull 1993). Obtained trees were visualised in FigTree version 1.4.3 (http://tree.bio.ed.ac.uk/software/figtree/). Bootstrap values (BS) ≥ 70% were considered significant. The ITS+nrLSU alignment was deposited in TreeBase (http://purl.org/phylo/treebase/phylows/study/TB2:S26552).

## ﻿Results

### ﻿Phylogenetic inferences

**Figure 1.**
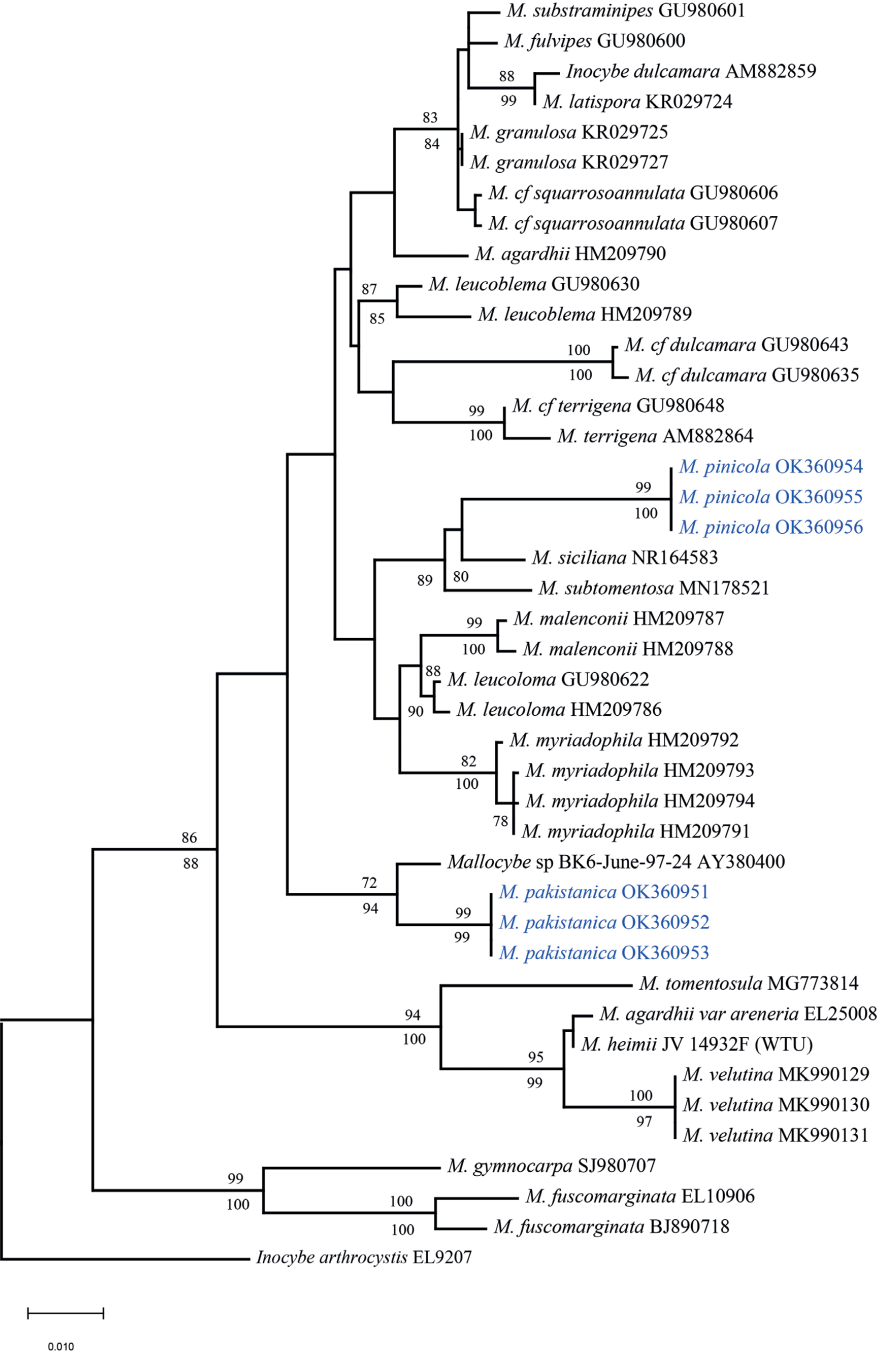
Phylogeny of *Mallocybepakistanica*, *M.pinicola* and related taxa produced from Maximum Likelihood (ML) inference using combined dataset of ITS and nrLSU sequences. Numbers on branches are ML/MP bootstrap values (only ≥ 70). New sequences reported in this study are blue coloured.

In this study, twelve novel sequences of two genes i.e. ITS and LSU were newly generated from our collections. Combined dataset I (ITS+LSU) contained forty-two sequences from eighteen taxa (Table 1), including six novel sequences from our collections. The length of the aligned dataset was 1,765 bp of which 1,303 characters are constant, 413 are variable and parsimony-uninformative and 300 parsimony-informative. All characters are of unord type and have equal weight. One equally parsimonious tree (TL = 802, CI = 0.678, RI = 0.818, RC = 0.555, HI = 0.322) was derived from the MP analysis. The topologies of ML and MP phylogenetic trees obtained using this dataset were practically the same; therefore, only the tree inferred from the ML analyses is shown (Fig. 1). Both ML and MP bootstraps strongly support the placement of the new species within *Mallocybe*. The *Mallocybe* species formed a monophyletic lineage with strong support (MLB = 99%). The sequences of our six collections formed two independent clades, which were respectively recognised and described as two new species: *Mallocybepakistanica* and *Mallocybepinicola*. *M.pakistanica* was sister to *Mallocybe* sp. BK 6-June-97-24 (MN178541) with high supports, implying that they are closely related to each other. Another species M. *pinicola* clustered with *Mallocybesiciliana*NR_164583 (Brugaletta et al. 2017) and *M.subtomentosa* (MN178521) with strong support (MPB = 89%). These two new taxa from Pakistan can be distinguished, based on molecular phylogenetic data, as well as morphology and ecology.

**Table 1.** Taxa of Mallocybe included in the molecular phylogenetic analyses.

Species	Specimen voucher/Isolate	Country	Accession numbers		Reference
			ITS	nrLSU	
*I.dulcamara*	EL59-05	Norway	GU980643	GU980643	Cripps et al. (2010)
*I.dulcamara*	CLC 1333	USA	GU980635	GU980635	Cripps et al. (2010)
*M.agardhii*	AB980912	Denmark	HM209790	HM209790	Vauras and Larsson (2011)
*M.arenaria*	EL25008	France	FN550937	FN550937	Ryberg et al. (2010)
*M.arthrocystis*	EL9207	Sweden	FN550941	FN550941	Ryberg et al. (2010)
*M.cf.squarrosoannulata*	CLC1566	Not given	GU980606	GU980606	Cripps et al. (2010)
*M.cf.squarrosoannulata*	EL120-08	Not given	GU980607	GU980607	Cripps et al. (2010)
*M.fulvipes*	EL99-07	Sweden	GU980600	GU980600	Cripps et al. (2010)
*M.fuscomarginata*	EL10906	Sweden	FN550940	FN550940	Ryberg et al. (2010)
*M.fuscomarginata*	BJ890718	Sweden	GU980656	GU980656	Cripps et al. (2010)
*M.granulosa*	SJ84030	Not given	KR029725	KR029725	Ariyawansa et al. (2015)
*M.granulosa*	EL138-09	Not given	KR029727	KR029727	Ariyawansa et al. (2015)
*M.granulosa*	EL138-09	Sweden	KR029727	KR029727	Ariyawansa et al. (2015)
*M.granulosa*	SJ84030	Sweden	KR029725	KR029725	Ariyawansa et al. (2015)
*M.gymnocarpa*	SJ980707	Sweden	AM882866	AM882866	Ryberg et al. (2008)
*M.heimii*	JV 14932F (WTU)	USA	–	AY380379	Matheny (2005)
*M.latispora*	EL190-08	Not given	KR029724	KR029724	Ariyawansa et al. (2015)
*M.leucoblema*	SM2324	Sweden	GU980630	GU980630	Cripps et al. (2010)
*M.leucoblema*	JV2898	Finland	HM209789	HM209789	Vauras and Larsson (2011)
*M.leucoloma*	EL41-07	Sweden	GU980622	GU980622	Cripps et al. (2010)
*M.leucoloma*	Ohenoja 880810	Svalbard	HM209786	HM209786	Vauras and Larsson (2011)
*M.malenconii*	JV23101	Finland	HM209787	HM209787	Vauras and Larsson (2011)
*M.malenconii*	PAM98941302	France	HM209788	HM209788	Vauras and Larsson (2011)
*M.myriadophylla*	EL121-08	Sweden	HM209792	HM209792	Vauras and Larsson (2011)
*M.myriadophylla*	JV19678	Finland	HM209793	HM209793	Vauras and Larsson (2011)
*M.myriadophylla*	JV5968	Finland	HM209794	HM209794	Vauras and Larsson (2011)
*M.myriadophylla*	JV19652	Finland	HM209791	HM209791	Vauras and Larsson (2011)
***M.pakistanica***	**MSM#0061**	**Pakistan**	**OK360951**	**OK392118**	**This paper**
***M.pakistanica***	**MSM#00132**	**Pakistan**	**OK360952**	**OK392119**	**This paper**
***M.pakistanica***	**MSM#0201**	**Pakistan**	**OK360953**	**OK392120**	**This paper**
***M.pinicola***	**MSM#0060**	**Pakistan**	**OK360954**	**OK392121**	**This paper**
***M.pinicola***	**MSM#00131**	**Pakistan**	**OK360955**	**OK392122**	**This paper**
***M.pinicola***	**MSM#0200**	**Pakistan**	**OK360956**	**OK392123**	**This paper**
*M.substraminipes*	K70-148	USA	GU980601	GU980601	Cripps et al. (2010)
*M.substraminipes*	EL12-08	USA	GU980601	GU980601	Cripps et al. (2010)
*M.terrigena*	EL24-08	USA	GU980648	GU980648	Cripps et al. (2010)
*M.terrigena*	EL11704	Sweden	AM882864	AM882864	Ryberg et al. (2008)
*M.tomentosula*	TENN:071837	USA	MG773814	MG773814	Unpublished
*M.velutina*	MSM # 0048	Pakistan	MK990129	MK999927	Saba and Khalid (2020)
*M.velutina*	MSM # 0049	Pakistan	MK990130	MK999928	Saba and Khalid (2020)
*M.velutina*	MSM # 00050	Pakistan	MK990131	MK999929	Saba and Khalid (2020)

### ﻿Taxonomy

#### ﻿ Mallocybe pakistanica

Taxon classificationFungiAgaricalesInocybaceae

Saba & Khalid sp. nov.

07C02AD2-3466-5ECA-8A73-F5BFE91418D7

MycoBank No: MB843490

Figs 2 , 3

##### Diagnosis.

Most similar to *Mallocybemyriadophylla* described from north-western Europe, but differs by the absence of a crowded lamellae, different pileal colouration and somewhat larger basidiospores. Phylogenetically separated from other species of *Mallocybe* due to unique ITS and LSU sequences.

##### Types

**. *Holotype***: PAKISTAN, Prov. Khyber Pakhtunkhwa, Mansehra, Chattar Plain, under *Pinuswallichiana* A. B. Jacks., 22 September 2013, *leg.* M. Saba & A.N. Khalid; MSM#0061 (ISL-F002); GenBank accession nos. OK360951 (ITS), OK392118 (nrLSU). ***Paratype***: Pakistan, Prov. Khyber Pakhtunkhwa, Mansehra, Chattar Plain, under *Pinuswallichiana*, 2 September 2015, *leg.* M. Saba & A.N. Khalid; MSM#00132 (ISL-F003); GenBank accession nos. OK360952 (ITS), OK392119 (nrLSU). Sep 2021, MSM#0201, (ISL-F004); GenBank accession nos. OK360953 (ITS), OK392120 (nrLSU).

##### Etymology.

Referring to the country where it was discovered.

##### Description.

***Pileus*** 19–24 mm diam., plane; margin deflexed in mature basidiomata, not splitting; surface dull, scaly, floccose, light brown (7.5YR6/4) or pale yellowish-brown (10YR7/4). ***Lamellae*** adnexed, subdistant, margin entire, regular, concolorous, moderate brown (7.5YR3/4) or strong brown (5YR4/6), one to two tiers of lamellulae or variable. ***Stipe*** 31–35 mm, central, equal, floccose, pale yellowish-brown (5YR8/8) or pale yellow (2.5Y9/4), cortina zone not seen; annulus absent. ***Context*** pale brown, tough, up to 2 mm thick. ***Odour*** not distinctive, somewhat fungoid. ***Taste*** not recorded.

***Basidiospores*** 7.4–13.2 × 5–6.6 µm [x = 10.8 × 5.9 µm, Q = 1.3–2.2], ellipsoid, ovoid, thin-walled, pale brown with yellowish contents in KOH. ***Basidia*** with yellowish necropigment, 25.7–36 × 6.8–10.3 µm, clavate, usually four-spored, thin-walled, hyaline in KOH; sterigmata 3.6–5.3 µm. ***Pleurocystidia*** absent. ***Cheilocystidia*** 14.8–31 × 9–15.8 µm, cylindrical, hyaline, in chains. ***Caulocystidia*** 38–43.6 × 5.5–7.0 µm, hyphal, yellowish-brown in KOH with clamp connections at base, thin-walled, abundant at the apex of stipe. ***Pileipellis*** hyphae cylindrical, pale brown in mass in KOH, 5–12 µm, thin-walled. ***Stipitipellis*** hyphae cylindrical, 6–10 µm, yellowish or olivaceous in KOH. All structures inamyloid**. *Clamp connections*** present.

##### Habitat.

Occurring in September, solitary, scattered on the forest floor in stands of Pinuswallichiana (Pinaceae).

##### Known distribution.

Currently known from Western Himalayas, Pakistan.

##### Notes.

*Mallocybepakistanica* can be characterised by small to medium-sized basidiomata, pale yellowish-brown or light brown pileus, ellipsoid basidiospores and catenate cheilocystidia (in chains). Based on the phylogenetic analysis (Fig. 1), conducted using the combined dataset ITS+LSU, indicate that the closet taxon is *Mallocybe* sp. BK 6-June-97-24 (MN178541) which is not yet published (Personal communications with P. Brandon Matheny). *Mallocybemyriadophylla* (Vauras & E. Larss.) Matheny & Esteve-Rav. is another closely related taxon which can be differentiated by different colouration of the pileus (when young pale brownish-grey, then grey brown, pale brown to brown, centre often darkest yellow-brown), presence of crowded lamellae and somewhat smaller phaseoliform basidiospores ((7.3–) 7.9–9.6 (–10.6) × (4.5–) 4.7–5.5 (–5.7) μm) (Vauras and Larsson 2011).

Another closely-related species in the adjacent clade is *Mallocybetomentosula* Matheny & Esteve-Rav., in Matheny, Hobbs & Esteve-Raventós which morphologically can be differentiated by the presence of a superior cortinate ring-zone, slightly smaller size of basidiospores and by its occurrence in eastern North America. Both ML and MP phylogenetic analyses also clearly support the identity of this new taxon as independent monophyletic clade.

**Figure 2.**
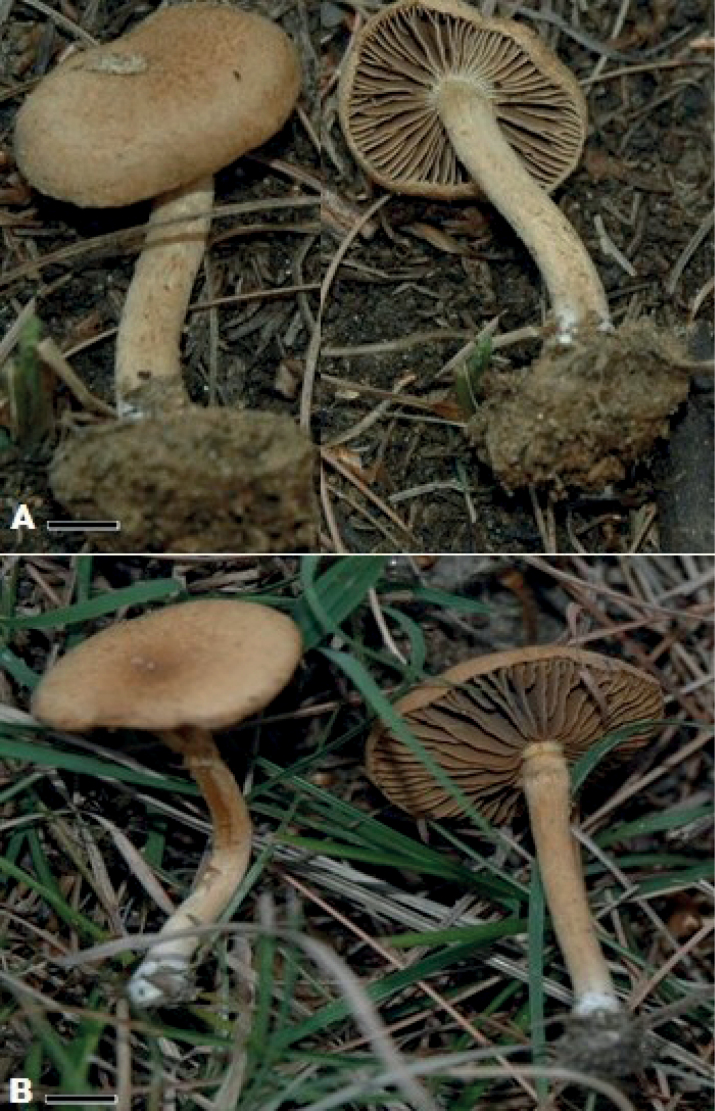
Holotypes **A***Mallocybepakistanica* (MSM#0061) **B***Mallocybepinicola* (MSM#0060). Scale bars: 10 mm (**A, B**).

**Figure 3.**
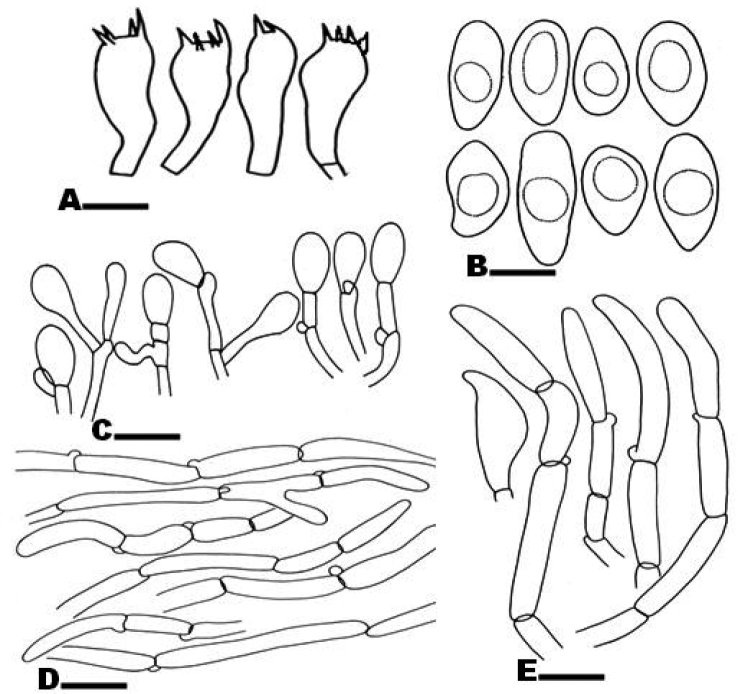
*Mallocybepakistanica* (holotype) **A** basidia **B** basidiospores **C** cheilocystidia **D** pileipellis **E** caulocystidia. Scale bars: 10 µm (**A**); 5 µm (**B**); 15 µm (**C**); 25 µm (**D**); 15 µm (**E**).

#### ﻿ Mallocybe pinicola

Taxon classificationFungiAgaricalesInocybaceae

Saba & Khalid sp. nov.

2AF83078-6673-54FD-97B4-58064C616930

MycoBank No: MB843491

Figs 2 , 4

##### Diagnosis.

Most similar to *M.siciliana* and *M.subtomentosa*, but differs by the combination of pileal colour, absence of umbo, size of basidiospores, pyriform to broadly clavate, catenate cheilocystidia and an ecological association with Pines. Phylogenetically separated from other species of *Mallocybe* due to unique ITS and LSU sequences.

##### Types.

***Holotype***: PAKISTAN, Prov. Khyber Pakhtunkhwa, Mansehra, Chattar Plain, under *Pinuswallichiana*, 22 September 2013, *leg.* M. Saba & A.N. Khalid; MSM#0060, (ISL-F005); GenBank accession nos. OK360954 (ITS), OK392121 (nrLSU). ***Paratype***: Paratype: Pakistan, Prov. Khyber Pakhtunkhwa, Mansehra, Chattar Plain, under *Pinuswallichiana*, 2 September 2015 *leg.* M. Saba & A.N. Khalid; MSM#00131(ISL-F006); GenBank accession nos. OK360955 (ITS), OK392122 (nrLSU). Sep 2021, MSM#0200, (ISL-F007); GenBank accession nos. OK360956 (ITS), OK392123 (nrLSU).

##### Etymology.

Referring to its exclusive association with *Pinus*.

##### Description.

***Pileus*** 24.9–27 mm diam., plan with slight depression in centre; margin straight or flaring, not splitting; surface dull, scaly, light orange (5YR8/8) or ochre-yellowish, central disc brownish-orange (5YR5/8). ***Lamellae*** adnexed, subdistant, margin eroded, strong brown (5YR4/6) or (5YR4/8). ***Stipe*** 31–35.6 mm, central, equal, floccose or pruinose near base, light orange (5YR8/8) or moderate orange (5YR7/8), cortina zone present; annulus absent. Context pale yellow to pale brown, tough, up to 3 mm thick. ***Odour*** faint not strong. ***Taste*** not recorded.

***Basidiospores*** (6.8–) 7.5–11 × 5–7 µm [x = 9.5 × 6.0 µm, Q = 1.1–1.8], ovoid, ellipsoid or phaseoliform, thin-walled, pale brown or golden brown in KOH. ***Basidia*** with yellowish necropigment, 27–42.4 × (5.4–) 8–12 µm, clavate, attenuated below, two to four-spored, thin-walled, hyaline in KOH; sterigmata 2.8–5.6 µm. ***Pleurocystidia*** absent. ***Cheilocystidia*** 11.8–36.5 × 11–15 µm, hyaline, pyriform to broadly clavate, in chains. ***Caulocystidia*** 22–70 × (6.3–) 7.7–14 µm, hyphal, yellowish-brown in KOH with clamp connections at base, thin-walled. ***Pileipellis*** hyphae cylindrical, hyaline singly or pale brown in mass in KOH, 5–11.3 µm, thin-walled, pileal hyphal endings 23.6–70 × 7.7–13 µm. ***Stipitipellis*** hyphae cylindrical, 5–10 µm, yellowish or olivaceous in KOH. All structures inamyloid. ***Clamp connections*** present.

##### Habitat.

Occurring in September, solitary, scattered on the forest floor in stands of Pinuswallichiana (Pinaceae).

##### Known distribution.

Currently known from Western Himalayas, Pakistan.

##### Notes.

*Mallocybepinicola* is characterised by light orange or ochre-yellowish, medium-sized pileus, absence of umbo, ovoid, ellipsoid or phaseoliform basidiospores, pyriform to broadly clavate, catenate cheilocystidia and its distribution in pine (conifer) forests. Based on the phylogenetic analysis (Fig. 1), constructed using the combined dataset of ITS and LSU, *M.pinicola* clustered with *M.siciliana* and *M.subtomentosa*. *M.siciliana* was originally described from Europe (Italy) by Brugaletta et al. (2017). It is similar to *M.siciliana* in having similar colour and size of pileus. However, it can be differentiated from *M.siciliana* by the entire absence of umbo and presence of larger basidiospores ((6.8–) 7.5–11 × 5–7 µm vs. 6.7–9 × 4.4–5.7 µm). Moreover, *Mallocybesiciliana* is described from forests having *Salix* species (*S.pedicellata* and *S.alba*), *Platunusorientalis*, *Tamarixgallica* and *Hypericumhircium*, while *M.pinnata* has been described from pure pine (*Pinuswallichiana*) forests.

Another closely-related taxa is *Mallocybesubtomentosa* which was originally described from the United States of America (Rouse’s Point). It resembles *M.pinicola* in having the entire absence of umbo, nearly similar spore size and shape of basidiospores (8–10 × 5–6 μm and ellipsoid basidiospores in *M.subtomentosa*). However, the presence of dark brown and minutely hairy to tomentose pileus, absence of cystidia and gregarious or subcaespitose habit in *M.subtomentosa* make the present species distinct from latter (Massee 1904).

Moreover, phylogenetic analysis (ML and MP), conducted using combined dataset of ITS + LSU, showed the clear separation of our species from these two closely-related taxa and all the sequences of our species clustered together with strong statistical support (99%) forming a monophyletic clade.

**Figure 4.**
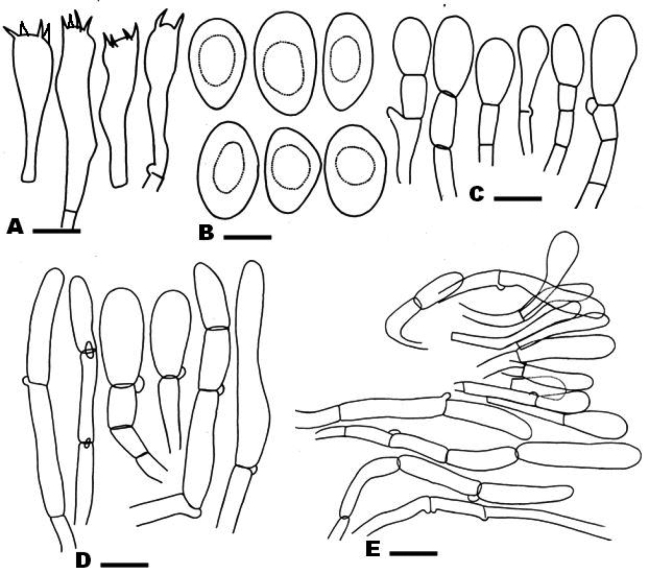
*Mallocybepinicola* (holotype) **A** basidia **B** basidiospores **C** cheilocystidia **D** caulocystidia **E** pileipellis. Scale bars: 10 µm (**A**); 5 µm (**B**); 15 µm (**C**); 20 µm (**D, E**).

## ﻿Discussion

Pakistan is located in southern Asia. This country is geographically diverse, ranging from the mountainous northern part, where the Himalayas meet their westernmost end, to the southern part with the coastal area along the Arabian Sea. Following the KöppenGeiger classification system for climate, 20 types can be found in Pakistan – including four arid, six temperate, eight cold and even two polar (Beck et al. 2018). Note that, despite this diversity in climate types, most of the country has a hot desert climate (Peel et al. 2007). Pakistan has a very rich flora; in an ongoing effort to compile the Flora of Pakistan, S.I. Ali and colleagues identified 5,521 plant species in 1,572 genera thus far (Ali 2008). When keeping the ratio between vascular plants and fungi (1:6) in mind (sensu Hawksworth 1991), this number of plants only hints at the true potential of in-depth mycological studies in Pakistan, which has been traditionally under-explored.

The multiple geographic features, different climates and plant species richness in Pakistan are suggestive of a high diversity of fungal species. In recent years, many papers have been published, describing new species from different fungal groups collected in Pakistan (e.g. Razaq et al. 2012; Nawaz et al. 2013; Thongklang et al. 2014; Qasim et al. 2015a, 2015b; Sarwar et al. 2015; Hussain et al. 2016, 2017, 2018; Jabeen et al. 2016; Farooqi et al. 2017; Naseer et al. 2018; Ullah et al. 2018; Saba et al. 2019a, 2019b; Kiran et al. 2020). Thirty-five species of *Inocybe* sensu lato have been reported from Pakistan (Ahmad et al. 1997; Ilyas et al. 2013; Saba et al. 2015; Jabeen et al. 2016; Farooqi et al. 2017; Razaq and Shahzad 2017; Naseer et al. 2018; Ullah et al. 2018; Song et al. 2019; this study). The genus *Mallocybe* is poorly known in Pakistan, with only two species that were known before this study: *M.leucoblema* (Kühner) Matheny & EsteveRav. and *M.velutina* Saba & Khalid (Ahmad et al. 1997; Saba and Khalid 2020).

In the combined ITS and LSU phylogenetic analysis, the new species described in this study occupy independent positions. From our morphological analysis, it is obvious that both *Mallocybepakistanica* and *M.pinicola* are separated from other closely-related *Mallocybe* species. With the contribution of this research work, the number of known taxa of this genus has been raised to sixty worldwide, with four from Pakistan. However, a considerable number of taxa have yet to be formally described and the number of the species will likely increase as more collections are studied from under-explored localities. A key to *Mallocybe* species reported from Pakistan is provided below;

**Table:** 

1	Basidiomata medium to large, pileus robust, ≥ 30 μm diam., cortina white	***M.leucoblema***
–	Basidiomata small to medium, pileus ≤ 30 μm diam., cortina brown	**2**
2	Pileus surface velutinous, cheilocystidia clavate or cylindrical	***M.velutina***
–	Pileus surface scaly, cheilocystidia articulated	**3**
3	Pileus light orange, moderate orange or brownish-orange, evenly coloured, plan, basidiospores longer and narrower	***M.pakistanica***
–	Pileus light orange or ochre-yellowish with central disc slightly depressed and brownish-orange, basidiospores smaller and broader	***M.pinicola***

## Supplementary Material

XML Treatment for Mallocybe pakistanica

XML Treatment for Mallocybe pinicola

## ﻿Additional information

### Author ORCIDs

Malka Saba https://orcid.org/0000-0001-7673-2345

Abdul Nasir Khalid https://orcid.org/0000-0002-5635-8031

Samina Sarwar https://orcid.org/0000-0002-6377-4498

### Data availability

All holotype and paratype collections of the new species are deposited at ISL. The sequences generated during this study are deposited in NCBI GenBank under accession numbers OK360951–OK360956 (ITS) and OK392118–OK392123 (LSU). The ITS þ nrLSU alignment is deposited in TreeBase (http://purl.org/phylo/treebase/phylows/study/TB2:S26552).
